# The fatty acid oleate is required for innate immune activation and pathogen defense in *Caenorhabditis elegans*

**DOI:** 10.1371/journal.ppat.1007893

**Published:** 2019-06-17

**Authors:** Sarah M. Anderson, Hilary K. Cheesman, Nicholas D. Peterson, J. Elizabeth Salisbury, Alexander A. Soukas, Read Pukkila-Worley

**Affiliations:** 1 Program in Innate Immunity, Division of Infectious Diseases and Immunology, University of Massachusetts Medical School, Worcester, MA, United States of America; 2 Center for Human Genomic Medicine and Diabetes Unit, Department of Medicine, Massachusetts General Hospital and Harvard Medical School, Boston, MA, United States of America; University of Texas Southwestern Medical Center at Dallas, UNITED STATES

## Abstract

Fatty acids affect a number of physiological processes, in addition to forming the building blocks of membranes and body fat stores. In this study, we uncover a role for the monounsaturated fatty acid oleate in the innate immune response of the nematode *Caenorhabditis elegans*. From an RNAi screen for regulators of innate immune defense genes, we identified the two stearoyl-coenzyme A desaturases that synthesize oleate in *C*. *elegans*. We show that the synthesis of oleate is necessary for the pathogen-mediated induction of immune defense genes. Accordingly, *C*. *elegans* deficient in oleate production are hypersusceptible to infection with diverse human pathogens, which can be rescued by the addition of exogenous oleate. However, oleate is not sufficient to drive protective immune activation. Together, these data add to the known health-promoting effects of monounsaturated fatty acids, and suggest an ancient link between nutrient stores, metabolism, and host susceptibility to bacterial infection.

## Author summary

The evolution of multicellular organisms has been shaped by their interactions with pathogenic microorganisms. The microscopic nematode *C*. *elegans* eats bacteria for food and has evolved inducible immune defenses toward ingested pathogens that are coordinated within intestinal epithelial cells. *C*. *elegans*, therefore, presents a genetic system to characterize the requirements for the activation of innate immune defenses. Here, we show that the monounsaturated fatty acid oleate is necessary for the induction of innate immune defenses and for protection against bacterial pathogens, which defines a new link between metabolism and the regulation of anti-pathogen responses in a metazoan host.

## Introduction

Fatty acids are the key structural components of phospholipids and triglycerides, and thereby affect nearly every facet of eukaryotic physiology. In addition to forming the building blocks of membranes and functioning as a currency of energy storage, fatty acid molecules promote health in a diverse number of ways. For example, fatty acids act as soluble signals for intracellular communication, affect membrane fluidity, and have been directly linked to lifespan regulation [1–3]. Conversely, excess stores of fatty acids in triglycerides lead to atherosclerosis and type 2 diabetes [4]. Thus, it is important to understand how individual fatty acids affect key physiological processes within a cell.

**Fig 1:**
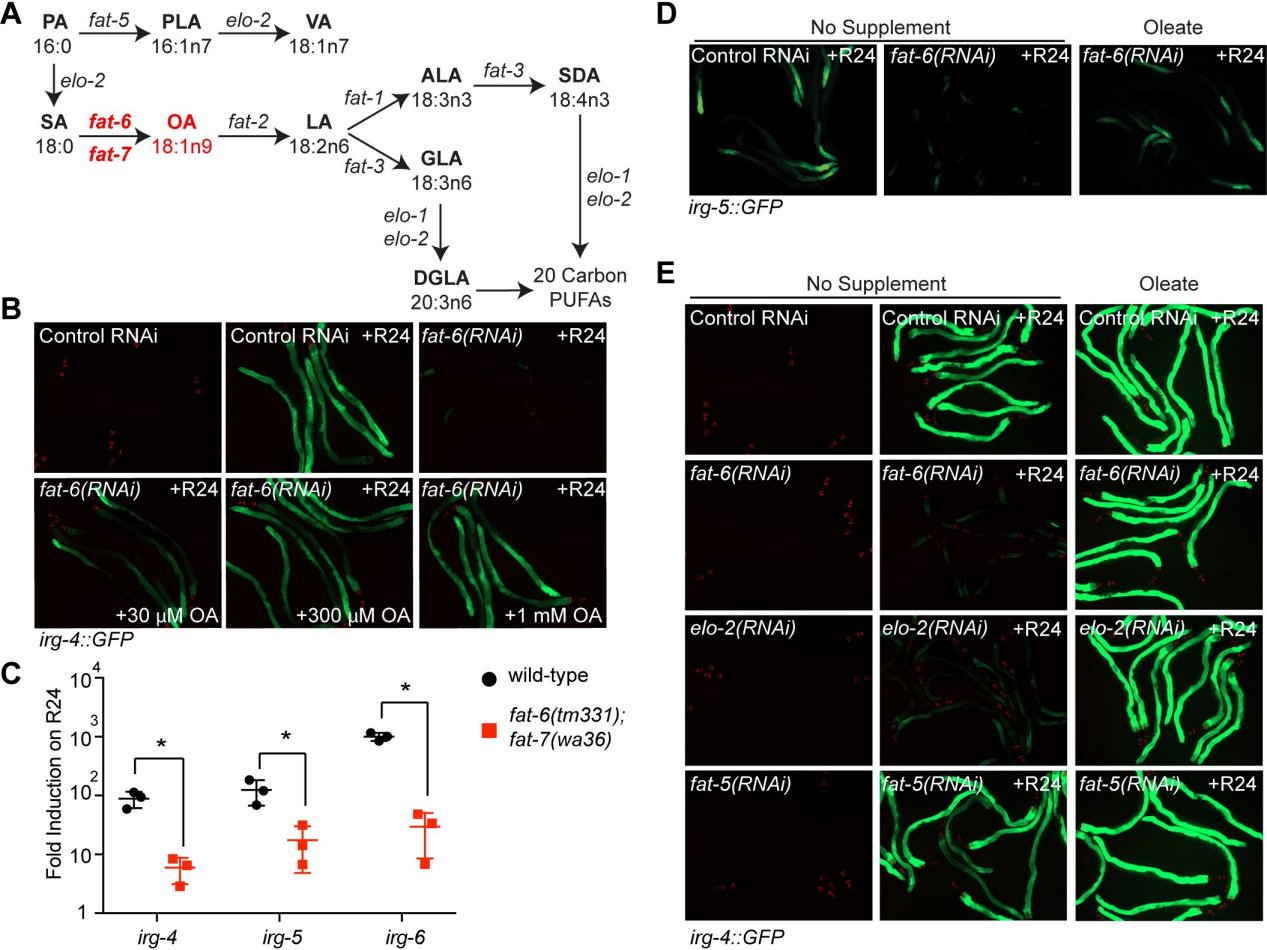
An RNAi screen identifies a role for MUFAs in the activation of *C*. *elegans* innate immune effectors. **A.** A schematic of the long-chain fatty acid synthesis pathway in *C*. *elegans* adapted from [6,17]. Fatty acid nomenclature: X:YnZ, X indicates the number of carbon atoms, Y denotes the number of double bonds, and Z designates the position of the terminal double bond relative to the methyl end of the molecule. Abbreviations: ALA, α-linoleic acid; DGLA, di-homo-γ-linoleic acid; GLA, γ-linoleic acid; LA, linoleic acid; OA, oleate; PA, palmitic acid; PLA, palmitoleic acid; SA, stearic acid; SDA, stearidonic acid; VA, vaccenic acid. **B.** The *C*. *elegans irg-4*::*GFP* immune reporter was exposed to control or *fat-6(RNAi)* bacteria seeded on plates containing control or the indicated levels of oleate. The animals were then transferred at the L4 stage to plates containing R24 for approximately 18 hours. Red pharyngeal expression is the *myo-2*::*mCherry* co-injection marker, which confirms the presence of the transgene. **C.** Expression of the immune effectors *irg-4*, *irg-5*, and *irg-6* were determined using qRT-PCR in wild-type or *fat-6(tm331);fat-7(wa36)* mutant animals exposed to R24 or solvent control (1% DMSO) for six hours. Data are the average of three independent replicates, each normalized to a control gene and presented as fold induction in R24-exposed vs. solvent control-exposed animals in the indicated genetic background. Error bars show standard deviation. Significance was determined by *t*-tests, * p<0.05. **D.** Animals containing the *irg-5*::*GFP* immune reporter were grown as described above and exposed to R24. All animals had a Rol phenotype, which confirms the presence of the transgene. **E.** *C*. *elegans* containing the *irg-4*::*GFP* immune reporter were fed RNAi bacteria targeting the indicated genes on plates containing control or oleate and exposed to R24, as described above.

The nematode *Caenorhabditis elegans* is a valuable model for studying the roles of fatty acids in metazoan biology [5–10]. Through the sequential action of conserved elongase (*elo*) and desaturase (*fat*) genes, nematodes can synthesize the full range of fatty acid molecules found in plants and animals [5–9]. Thus, *C*. *elegans* does not have a dietary requirement for specific fatty acids, unlike mammals. In nematodes, as in mammals, the majority of fatty acid molecules are synthesized from stearic acid, a saturated, 18-carbon molecule, which is progressively desaturated and elongated to a variety of monounsaturated (MUFA) and polyunsaturated (PUFA) fatty acids (Fig 1A) [5–9]. The contribution of individual fatty acids to specific biological processes can be characterized using genetic approaches in *C*. *elegans* at the level of an entire organism.

Nematodes rely on inducible host defense mechanisms to provide protection from ingested pathogens [11–14]. Because worms normally eat bacteria for food, their evolution has been shaped by interactions with both pathogenic and nonpathogenic microorganisms. The immune effectors in *C*. *elegans* include a suite of secreted proteins, including lysozymes, proteins with CUB-like domains, and ShK toxins, some of which are required for host defense during bacterial infection [15–18]. *C*. *elegans* with mutations that abrogate the induction of these immune effectors during infection are hypersusceptible to killing by bacterial pathogens [15,19,20]. In this study, we define a requirement for the MUFA oleate in innate immune activation and pathogen defense in *C*. *elegans*. Previously, Nandakumar et al. showed that two polyunsaturated fatty acids, γ-linolenic acid (GLA) and stearidonic acid (SDA), are required for the basal expression of innate immune effectors [17]. Here, we show that oleate is necessary for innate immune activation and resistance to bacterial infection in a manner distinct from the effects of GLA and SDA. Because oleate is among the most abundant fatty acids in cells, our data suggest an ancient link between cellular energy stores and immune activation.

## Results

### An RNAi screen identifies a role for MUFAs in the activation of *C*. *elegans* innate immune effectors

We previously conducted an RNAi screen of 1,420 intestinal genes for innate immune regulators in *C*. *elegans* [21]. We used an immunostimulatory small molecule called R24 (also called RPW-24) and a GFP-based transcriptional reporter, *irg-4*::*GFP*, which provides a convenient readout of innate immune activation [18,21–25]. The gene *irg-4* (F08G5.6) is transcriptionally upregulated during infection with multiple pathogens and contains a CUB-like domain, which is present in many of the secreted immune effectors in *C*. *elegans* [15,16,18]. *irg-4* is required for normal resistance to bacterial infection, but does not modulate the normal lifespan of *C*. *elegans* or affect susceptibility to other stressors [16–18]. *irg-4* is also strongly upregulated by R24, a xenobiotic that protects nematodes from bacterial infection by boosting innate immune responses [18,21,22,25]. Because of its potent immunostimulatory properties, R24 is a useful tool for dissecting the metabolic requirements of immune activation without altering the bacterial diet of *C*. *elegans* [18,21–25]. The RNAi screen identified 29 gene inactivations that are required for the R24-mediated induction of the innate immune reporter *irg-4*::*GFP* [21]. Interestingly, two of the genes identified in this screen are the stearoyl-coenzyme A (CoA) desaturases *fat-6* and *fat-7*, which function redundantly to synthesize oleate from stearic acid (Fig 1A) [5]. RNAi-mediated knockdown of *fat-6* completely abrogated the induction of *irg-4*::*GFP* by R24 (Fig 1B) while knockdown of *fat-7* partially suppressed its upregulation (S1A Fig).

To validate the results of the RNAi studies in the *irg-4*::*GFP* transcriptional reporter, we used qRT-PCR to examine the transcriptional regulation of the *irg-4* gene in wild-type and in *fat-6(tm331);fat-7(wa36)* double-mutant animals, which are deficient in oleate production [5,8]. The R24-mediated induction of *irg-4* was reduced in *fat-6(tm331);fat-7(wa36)* animals compared to wild-type animals (Fig 1C). In addition, the induction of two other immune effectors, *irg-5*(F35E12.5) and *irg-6*(C32H11.1), was also attenuated in the *fat-6(tm331);fat-7(wa36)* double mutant (Fig 1C). Like *irg-4*, *irg-5* and *irg-6* are strongly induced during infection with the bacterial pathogen *P*. *aeruginosa* and by the immunostimulatory xenobiotic R24 [18,21–23]. In addition, knockdown of *irg-5* and *irg-6* makes *C*. *elegans* more susceptible to infection by *P*. *aeruginosa* [18]. The defect in immune activation by *fat-6(RNAi)* was also visualized using the *irg-5*::*GFP* transcriptional reporter (Fig 1D). Thus, stearoyl-CoA desaturases are required for the induction of at least three key immune effectors in *C*. *elegans*.

We performed fatty acid supplementation experiments to determine if the effect of the stearoyl-CoA desaturases on immune activation depends specifically on the production of the MUFA oleate. Interestingly, supplementation of exogenous oleate rescued, in a dose-dependent manner, the R24-mediated immune activation defect of the *irg-4*::*GFP* reporter strain in *fat-6(RNAi)* animals (Fig 1B). Oleate is also required for the upregulation of *irg-5*::*GFP* by R24, as supplementation of this MUFA rescued the induction defect conferred by knockdown of *fat-6* (Fig 1D).

Consistent with a key role for MUFAs in immune activation, knockdown of the elongase *elo-2*, which catalyzes the conversion of palmitic acid to stearic acid, the step immediately upstream of oleate synthesis, also suppressed the activation of *irg-4*::*GFP* by R24 (Fig 1E). Importantly, oleate supplementation also fully complemented the immune activation defect of *elo-2(RNAi)* animals (Fig 1E). These data demonstrate that lack of oleate, and not an accumulation of upstream stearic acid, is responsible for deficits in immune effector induction. In addition, knockdown of *fat-5*, the palmitoyl-CoA desaturase, which also preferentially acts on palmitic acid, but converts it to a different MUFA, palmitoleic acid (PLA), had no effect on the induction of *irg-4*::*GFP* (Fig 1E).

Our RNAi screen also identified the mediator subunit *mdt-15* among the 29 gene inactivations that are required for the R24-mediated induction of the innate immune reporter *irg-4*::*GFP* [21]. We subsequently showed that *mdt-15* is required for the induction of innate immune effectors and for defense against the bacterial pathogen *Pseudomonas aeruginosa* [21]. In addition to its role as an immune regulator, MDT-15 controls the transcription of a suite of fatty acid biosynthesis enzymes [26–28]. Interestingly, oleate supplementation did not rescue the induction of *irg-4*::*GFP* in *mdt-15(tm2182)* loss-of-function animals, indicating that *mdt-15* controls multiple steps in the activation of innate immune effectors (S1B Fig).

### Polyunsaturated fatty acids (PUFAs) are not required for immune effector induction by the immunostimulatory xenobiotic R24

**Fig 2:**
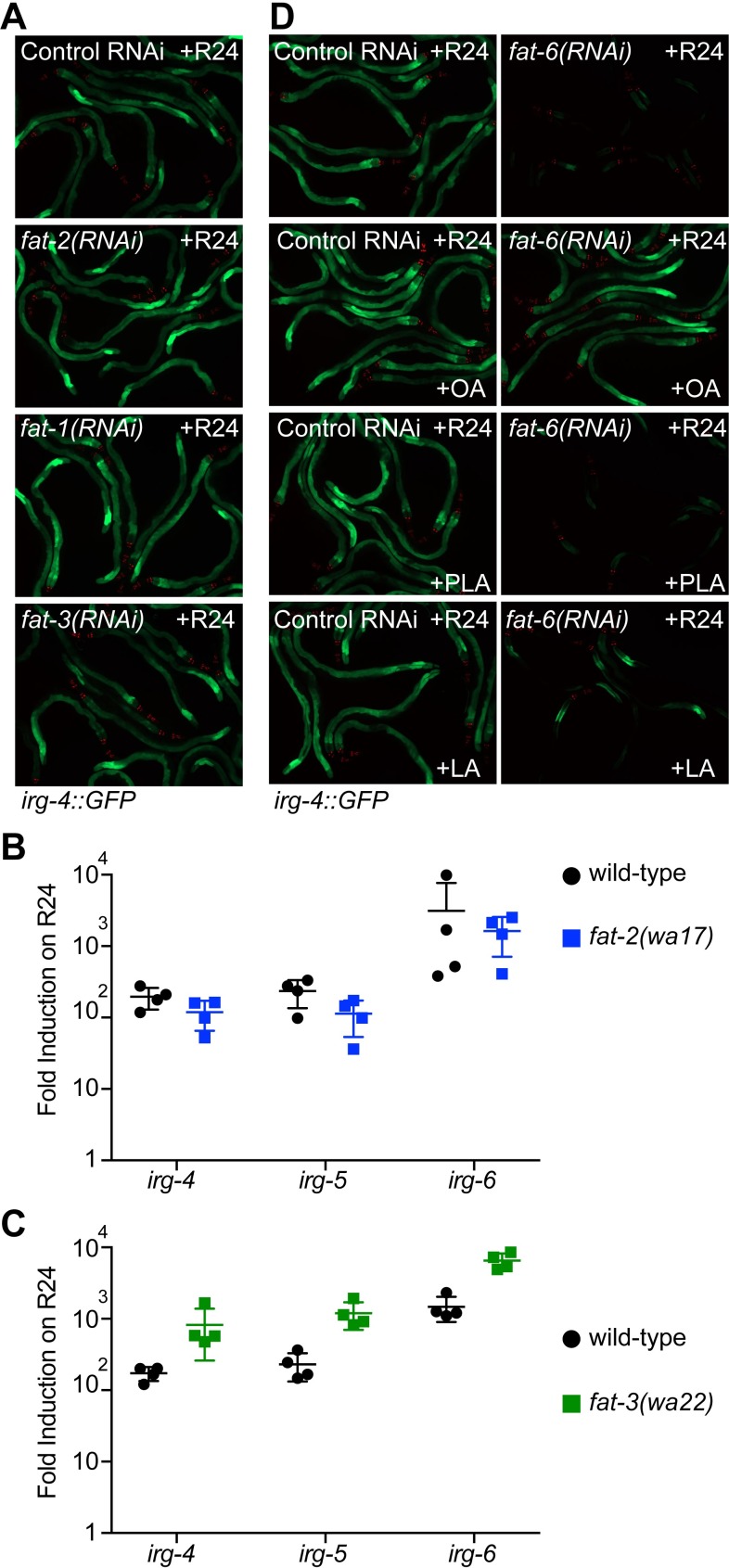
Polyunsaturated fatty acids are not required for immune induction by the immunostimulatory small molecule R24. **A.** *C*. *elegans irg-4*::*GFP* immune reporter animals were fed the indicated RNAi bacteria and transferred at the L4 stage to plates containing R24 for approximately 18 hours. **B and C.** Expression of the immune effectors *irg-4*, *irg-5*, and *irg-6* were determined using qRT-PCR in wild-type, *fat-2(wa17)*, and *fat-3(wa22)* mutant animals exposed to R24 or the solvent control (DMSO) for six hours. Data are the average of four independent replicates, each normalized to a control gene and presented as fold induction in R24-exposed vs. solvent control-exposed animals in the indicated genetic background. Error bars show standard deviation. Significance was determined by *t*-tests. There was no significant difference in the R24-mediated induction of the indicated gene in *fat-2(wa17)* animals or in *fat-3(wa22)* mutants for *irg-4*. In *fat-3(wa22)* animals, the induction of *irg-5* and *irg-6* by R24 was significantly higher than in wild-type (p <0.001). **D.** *C*. *elegans irg-4*::*GFP* immune reporter animals were fed control or *fat-6(RNAi)* bacteria seeded on plates containing control or 500 μM of the indicated fatty acid. PLA is palmitoleic acid and LA is linoleic acid. The animals were then transferred at the L4 stage to plates containing R24 for approximately 18 hours. The presence of the transgene is confirmed by red pharyngeal expression of the *myo-2*::*mCherry* co-injection marker.

Monounsaturated fatty acids are converted to polyunsaturated fatty acids (PUFAs) by desaturases that initially use oleate as a substrate [6]. To determine if a PUFA is required for immune effector induction by the immunostimulatory xenobiotic R24, we used both genetic and fatty acid complementation experiments. We examined the induction of *irg-4*::*GFP* in animals deficient in the desaturase *fat-2*, which catalyzes the first step in PUFA synthesis (the conversion of oleate to linoleic acid), and also *fat-1(RNAi)* and *fat-3(RNAi)*, the enzymes that act downstream of *fat-2* in the synthesis of PUFAs (Fig 1A) [6,7]. Knockdown of *fat-1*, *fat-2*, or *fat-3* had no effect on the induction of *irg-4*::*GFP* by R24 (Fig 2A). We confirmed this RNAi experiment using the *fat-2(wa17)* and the *fat-3(wa22)* loss-of-function mutants (Fig 2B and 2C). Notably, the R24-mediated induction of the immune effectors *irg-4*, *irg-5*, and *irg-6* in the *fat-2(wa17)* and the *fat-3(wa22)* mutants were not significantly lower than in wild-type animals (Fig 2B and 2C).

Supplementation of individual fatty acids to *fat-6(RNAi)* animals confirmed these genetic observations. Unlike oleate supplementation, addition of the 16 carbon MUFA palmitoleic acid (PLA), which is synthesized by the desaturase *fat-5* (Fig 1A), did not complement the *irg-4*::*GFP* induction defect in *fat-6(RNAi)* animals (Fig 2D). In addition, supplementation of the PUFA linoleic acid (LA), which is synthesized by *fat-2* using oleate as a substrate (Fig 1A), also failed to fully complement the *irg-4*::*GFP* induction defect of *fat-6(RNAi)* animals (Fig 2D). Together, these data show that the fatty acid oleate, and not another MUFA or PUFA, is required for the induction of the innate immune effector genes by an immunostimulatory small molecule.

### Stearoyl-CoA desaturase activity is required for immune effector induction

**Fig 3:**
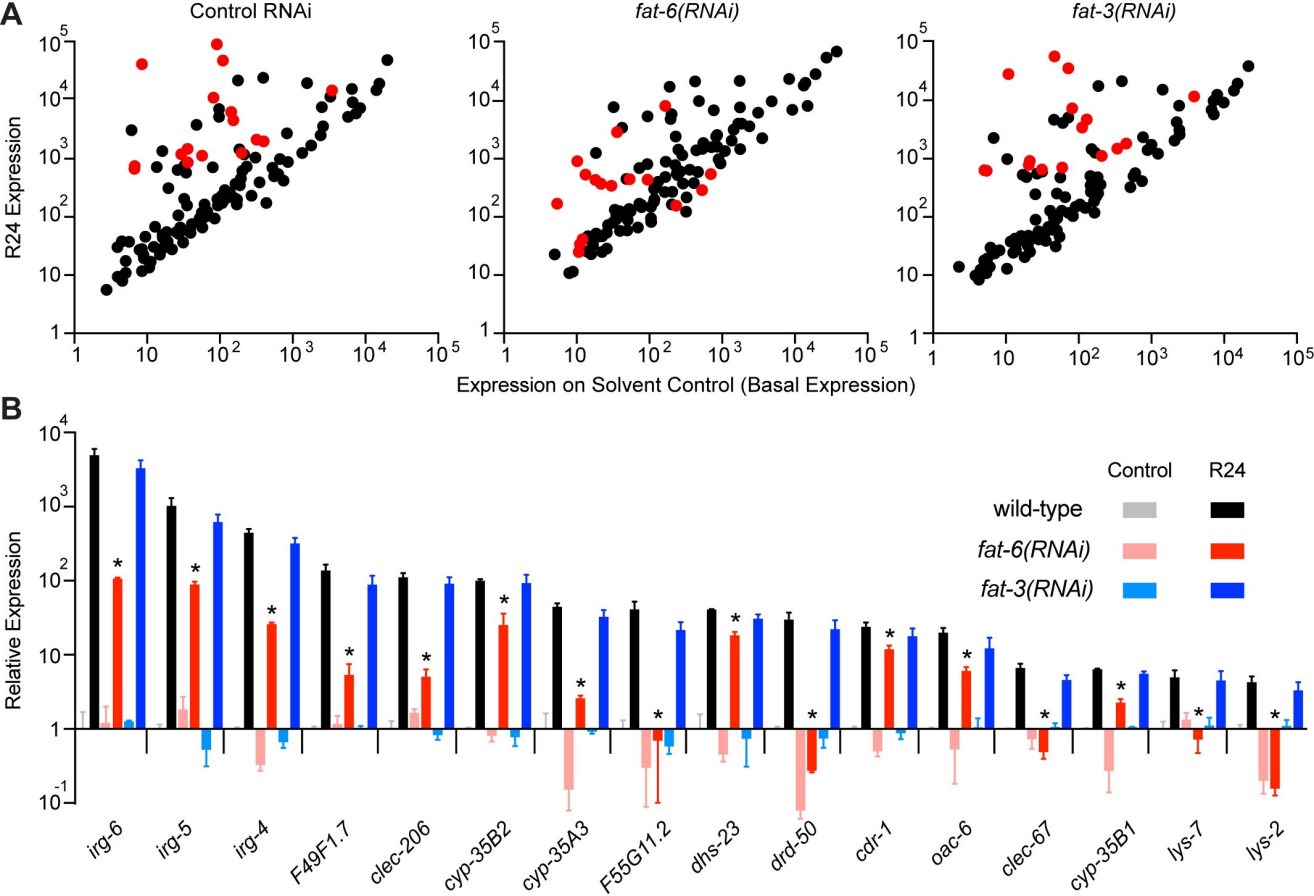
Stearoyl-CoA desaturase activity is required for the induction of an innate immune response in *C*. *elegans*. **A.** Dot plots showing the NanoString nCounter gene expression analysis of 118 immune and stress response genes in *C*. *elegans* grown on the indicated RNAi bacteria and exposed to control or R24. Each dot represents one gene. Red dots highlight the 16 genes whose R24-mediated induction is significantly suppressed in *fat-6(RNAi)* animals. Data are the average of two independent replicates. **B.** The expression of the 16 *fat-6*-dependent genes in the indicated genotypes and conditions is shown relative to wild-type animals exposed to control. Significance was determined using *t*-tests, * p<0.05. Data are the average of two independent replicates, each normalized to three control genes and expressed relative to the baseline condition (wild-type animals exposed to control) for each gene. Error bars show standard deviation.

To determine if stearoyl-CoA desaturase activity has a broad effect on the induction of innate immune effectors, we profiled the transcription of 118 immune and stress response genes in wild-type, *fat-6(RNAi)*, and *fat-3(RNAi)* animals, each exposed to the solvent control (DMSO) or R24 (Fig 3A). Of the 40 genes that were induced at least 4-fold by R24, the upregulation of 16 genes was significantly attenuated in *fat-6(RNAi)* animals (Fig 3A and 3B and S1 Table). As we observed in our studies of *irg-4*, *irg-5*, and *irg-6* in the *fat-3(wa22)* mutant (Fig 2C), the induction of these 16 *fat-6*-dependent genes was not affected by knockdown of *fat-3* (Fig 3A and 3B). For this transcription profiling experiment, we chose to use *fat-6(RNAi)* to examine the effects of oleate depletion on immune activation. Others have also used single knockdown of either *fat-6* or *fat-7* to recapitulate the phenotypes observed in the *fat-6(tm331);fat-7(wa36)* double mutant [3,29]. Of note, the R24-mediated upregulation of *irg-4*, *irg-5*, and *irg-6* was not attenuated in the *fat-6(tm331)* or *fat-7(wa36)* single mutants (S2A and S2B Fig), but the induction of these immune effectors was suppressed in *fat-6(RNAi)* animals (Fig 3B), as in the *fat-6(tm331);fat-7(wa36)* double mutants (Fig 1C). We also confirmed by gas chromatography-mass spectrometry (GC-MS) that knockdown of *fat-6* significantly decreases the pool of oleate and causes accumulation of the upstream fatty acid stearate (S1C Fig).

Interestingly, each of the 16 *fat-6*-dependent, *fat-3*-independent genes encode putative immune effectors that are induced during infection with at least one bacterial pathogen, a group that includes the innate immune effectors *irg-4*, *irg-5*, and *irg-6* (Fig 3B). Twenty-four genes, however, were induced by R24 in a manner independent of *fat-6* (S1 Table). Thus, *fat-6* modulates the transcription of a specific subset of genes, including a group of innate immune effectors. Moreover, the observation that the induction of these 16 genes was not controlled by *fat-3* further supports the specificity of *fat-6* in the regulation of innate immune responses.

### Oleate is required for host resistance to *P*. *aeruginosa* infection

**Fig 4:**
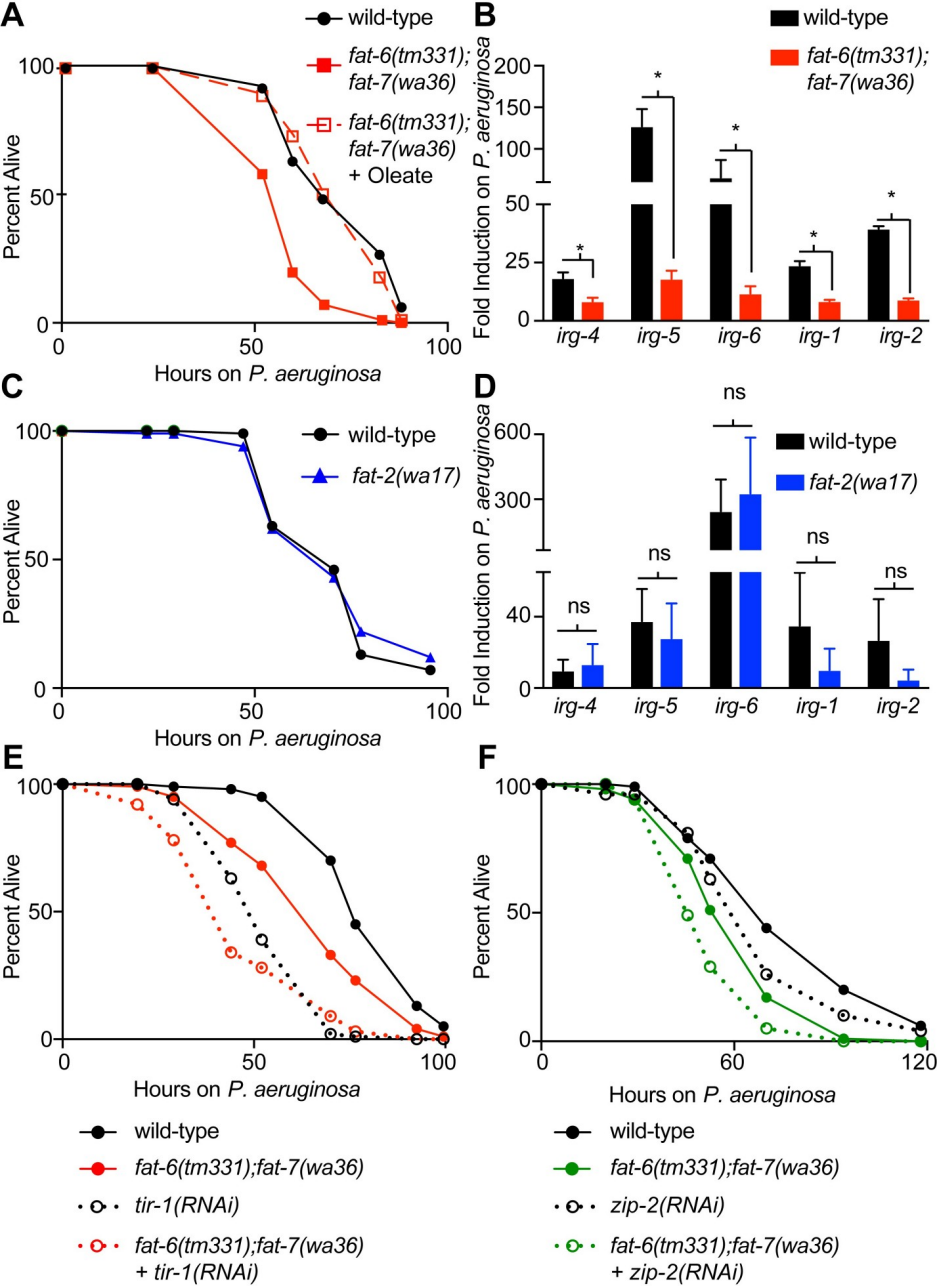
Oleate is required for host resistance to *P*. *aeruginosa* infection. **A and C.** *P*. *aeruginosa* pathogenesis assays of wild-type and *fat-6(tm331);fat-7(wa36)* (A) or *fat-2(wa17)* (C) animals grown on control media or oleate-supplemented media, as indicated. Animals were transferred at the L4 stage to plates containing *P*. *aeruginosa*. Assay plates were not supplemented with oleate. *fat-6(tm331);fat-7(wa36)* animals are more susceptible to killing by *P*. *aeruginosa* (p<0.01). Oleate supplementation rescues the enhanced susceptibility to pathogen phenotype of the *fat-6(tm331);fat-7(wa36)* mutant (p<0.01). *fat-2(wa17)* animals are not hypersusceptible to killing (p = not significant). Data are representative of at least two trials. Sample sizes, mean lifespan, and p values for all trials are shown in S2A Table. Significance was determined using Kaplan-Meier survival curves and log-rank tests. **B and D.** Expression of the indicated genes measured by qRT-PCR in wild-type, *fat-6(tm331);fat-7(wa36)* double-mutant, *or fat-2(wa17)* animals exposed to *P*. *aeruginosa* at the L4 stage for 6 hours. Data are the average of three independent replicates, each normalized to a control gene and presented as fold induction in animals of the indicated genetic background infected with *P*. *aeruginosa* vs. animals fed the standard bacterial food source (*E*. *coli* OP50). Error bars show standard deviation. Statistical significance was determined using t-tests. * p<0.05. **E and F.** *P*. *aeruginosa* pathogenesis assays of wild-type and *fat-6(tm331);fat-7(wa36)* exposed to control RNAi bacteria, *tir-1(RNAi)* or *zip-2(RNAi)* bacteria, as indicated. *fat-6(tm331);fat-7(wa36)+ tir-1(RNAi)* animals are significantly more susceptible than *fat-6(tm331);fat-7(wa36)* mutants and *tir-1(RNAi)* animals (p<0.05). In addition, *fat-6(tm331);fat-7(wa36)+ zip-2(RNAi)* animals are significantly more susceptible than *fat-6(tm331);fat-7(wa36)* mutants and *zip-2(RNAi)* animals (p<0.05). Data are representative of at least two trials. Sample sizes, mean lifespan, and p values for all trials are shown in S2A Table. Significance was determined using Kaplan-Meier survival curves and log-rank tests.

Of the 16 genes whose R24-mediated induction was dependent on *fat-6*, ten are putative immune effectors that are also induced during infection with *P*. *aeruginosa*, including three known modulators of the host susceptibility to pseudomonal infection, *irg-4*, *irg-5*, and *irg-6* [16–18]. To determine if oleate is important for host defense in *C*. *elegans*, we performed pathogenesis assays with *P*. *aeruginosa*. The *fat-6(tm331);fat-7(wa36)* double mutant was more susceptible to infection by *P*. *aeruginosa* than wild-type animals, consistent with a prior report [17] (Fig 4A and S2A Table). Importantly, *fat-6(tm331);fat-7(wa36)* animals have a similar lifespan as wild-type animals when grown under standard laboratory conditions [30]. These data suggest that the hypersusceptibility to pathogen-mediated killing in the *fat-6(tm331);fat-7(wa36)* double mutant is not secondary to pleiotropic effects of these mutations on worm fitness. Supplementation of oleate to the *fat-6(tm331);fat-7(wa36)* animals fully complemented the enhanced susceptibility of this mutant to pathogen infection (Fig 4A and S2A Table). Of note, *fat-6(tm331)* and *fat-7(wa36)* single mutant worms are not more susceptible to pathogen-mediated killing than wild-type animals, as noted previously [17] (S3 Fig and S2B Table). Consistent with the key role of *fat-6* and *fat-7* in the regulation of innate immune responses, the fold induction of the innate immune effectors *irg-4*, *irg-5*, *irg-6*, *irg-1*, and *irg-2* during pseudomonal infection was significantly attenuated in the *fat-6(tm331);fat-7(wa36)* double mutant compared to wild-type (Fig 4B).

Nandakumar et al. previously defined a role for two PUFAs, GLA and SDA, in the basal regulation of innate immune effectors and pathogen resistance in *C*. *elegans* [17]. We considered whether the effect of oleate on the activation of immune responses could occur through its metabolism to GLA and SDA; however, several lines of evidence show that this is not the case. The pathogen susceptibility and immune effector transcription profile of *fat-2(wa17)* mutants demonstrate that the effect of oleate on innate immune activation is not dependent on the production of PUFAs. The desaturase *fat-2* acts immediately downstream of oleate production to catalyze the first step in PUFA biosynthesis (Fig 1A). *fat-2(wa17)* mutant animals are not more susceptible to *P*. *aeruginosa* infection than wild-type animals (Fig 4C and S2A Table). In addition, the induction of the immune effectors *irg-4*, *irg-5*, *irg-6*, *irg-1*, and *irg-2* during pseudomonal infection was not compromised in *fat-2(wa17)* mutants compared to wild-type (Fig 4D), unlike what we observed for the *fat-6(tm331);fat-7(wa36)* double mutant (Fig 4B). We also examined the desaturase *fat-3*, which acts downstream of *fat-2* in the synthesis of PUFAs, including GLA and SDA (Fig 1A). Nandakumar et al. previously showed that *fat-3* is required for resistance to *P*. *aeruginosa* via the fatty acids GLA and SDA [17]. We found that exogenous oleate did not rescue the enhanced susceptibility of the *fat-3(wa22)* mutant to pseudomonal infection (S4 Fig and S2D Table). Thus, *fat-6* and *fat-7* affect pathogen susceptibility specifically through the production of oleate, in a manner that is independent of PUFA synthesis via the enzymes *fat-2* or *fat-3*.

Stearoyl-CoA desaturases are required for the induction of immune effectors, such as *irg-4*, *irg-5*, and *irg-6*, whose basal, or resting, expression is dependent on the p38 MAPK PMK-1 innate immune pathway and those, like *irg-1* and *irg-2*, whose transcription are regulated independent of this canonical immune pathway (Fig 4B) [15,18,21,31]. Interestingly, knockdown of *tir-1*, the Toll/IL-1 (TIR) domain protein that is an integral component the p38 MAPK PMK-1 signaling cassette [19,32,33], further enhanced the susceptibility of the *fat-6(tm331);fat-7(wa36)* double loss-of-function mutant strain to pseudomonal infection (Fig 4E and S2A Table). Likewise, knockdown of the bZIP transcription factor *zip-2*, which controls the induction of *irg-1* and *irg-2* during *P*. *aeruginosa* infection [31], caused the *fat-6(tm331);fat-7(wa36)* double mutant to be more susceptible to killing by *P*. *aeruginosa* (Fig 4F and S2A Table). These data suggest that *fat-6* and *fat-7* are required for the proper expression of a broad group of innate immune effectors via a mechanism that operates in parallel to the p38 MAPK PMK-1 and ZIP-2 immune pathways.

### Stearoyl-CoA desaturase activity is necessary for protection against diverse bacterial pathogens

**Fig 5:**
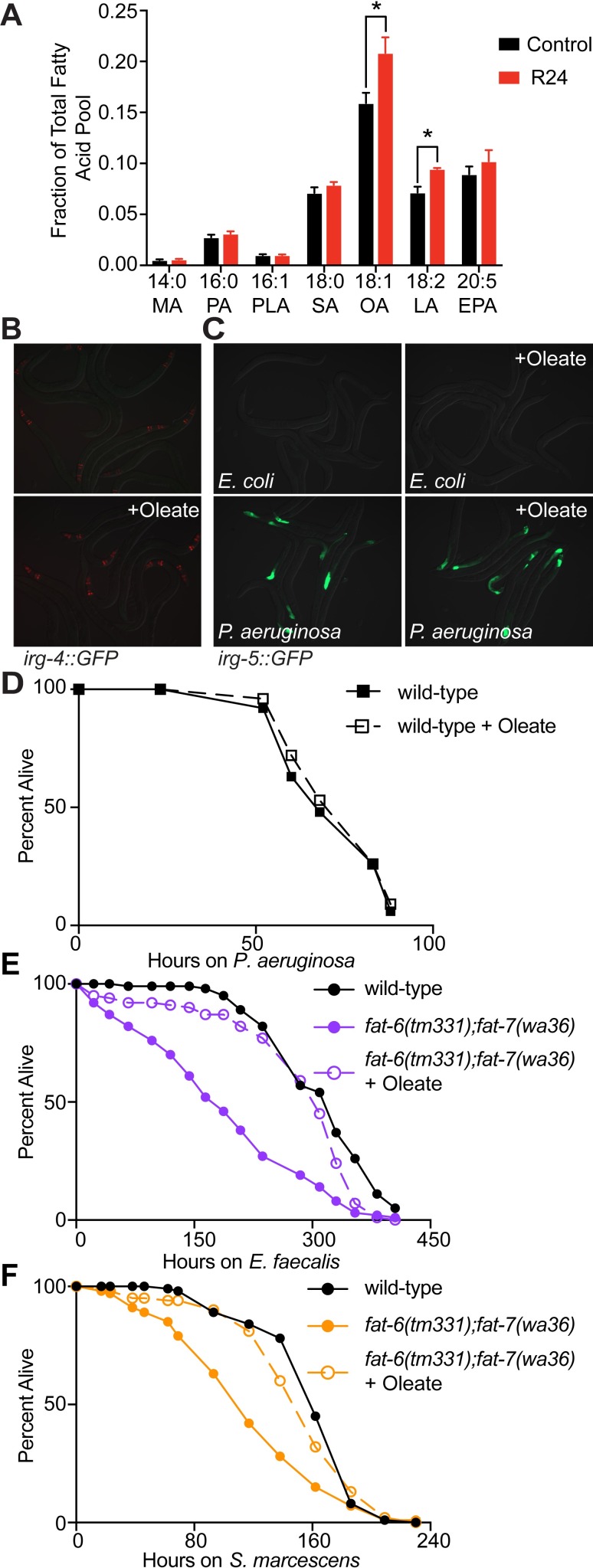
Oleate is necessary but not sufficient for pathogen resistance in *C*. *elegans*. **A.** GC-MS of L4-stage animals exposed to R24 or solvent control for 24 hours. Data are the average of three independent replicates with error bars showing standard deviation. Statistical analyses performed using two-way ANOVA with Bonferroni correction. * p<0.05. Abbreviations: MA, myristic acid; PA, palmitic acid; PLA, palmitoleic acid; SA, stearic acid; OA, oleic acid; LA, linoleic acid; EPA, eicosapentaenoic acid. **B and C.** *C*. *elegans irg-4*::*GFP* (B) or *irg-5*::*GFP* (C) animals were grown on plates containing control or oleate, as indicated. The animals were then transferred at the L4 stage to plates containing *P*. *aeruginosa* or *E*. *coli* OP50, as indicated. **D, E, F.** *C*. *elegans* pathogenesis assays with *P*. *aeruginosa* (D), *E*. *faecalis* (E) and *S*. *marcescens* (F) in wild-type and *fat-6(tm331);fat-7(wa36)* animals grown on control media or oleate-supplemented media, as indicated. Animals were transferred at the L4 stage to plates containing the indicated pathogen. Assay plates were not supplemented with oleate. There is no significant difference between the conditions in D. In E and F, the *fat-6(tm331);fat-7(wa36)* mutants are more susceptible to killing by the indicated pathogen (p<0.01). Oleate supplementation rescues the enhanced susceptibility to pathogens phenotype of the *fat-6(tm331);fat-7(wa36)* mutant in both E and F (p<0.01). Data are representative of at least two trials. Sample sizes, mean lifespan, and p values for all trials are shown in S2C Table. Significance was determined using Kaplan-Meier survival curves and log-rank tests.

We performed GC-MS to determine if R24 treatment changes the abundance of cellular oleate. Interestingly, GC-MS revealed that the fraction of both oleate and linoleic acid relative to the total fatty acid pool significantly increased in R24-treated samples compared to controls (Fig 5A). Together, these data show that treatment with the immunostimulatory xenobiotic R24 shifts the fatty acid pool towards more oleate.

Because oleate is required for the induction of innate immune effectors and is increased in the presence of R24, we asked if this MUFA is sufficient for innate immune activation in *C*. *elegans*. However, the addition of oleate to the standard bacterial food source for *C*. *elegans* did not activate GFP expression in the *irg-4*::*GFP* or the *irg-5*::*GFP* transcriptional reporters (Fig 5B and 5C). The presence of oleate in the growth media also did not further augment the induction of *irg-5*::*GFP* during *P*. *aeruginosa* infection (Fig 5C). In addition, oleate treatment did not extend the lifespan of wild-type *C*. *elegans* during *P*. *aeruginosa* infection (Fig 5D and S2C Table). Thus, oleate is necessary, but not sufficient, for immune activation and resistance to *P*. *aeruginosa* infection in *C*. *elegans*.

Interestingly, *fat-6(tm331);fat-7(wa36)* mutant animals were also hypersusceptible to infection with the gram-positive pathogen *Enterococcus faecalis* (Fig 5E and S2C Table) and *Serratia marcescens* (Fig 5F and S2C Table), which, like *P*. *aeruginosa*, is a gram-negative bacteria. Importantly, the enhanced susceptibility of the *fat-6(tm331);fat-7(wa36)* mutants to infection with *E*. *faecalis* and *S*. *marcescens* was rescued by treatment with exogenous oleate (Fig 5E and 5F). Thus, oleate is required for host resistance to diverse bacterial pathogens.

## Discussion

This study defines a role for the MUFA oleate in *C*. *elegans* innate immune activation. We show that animals deficient in oleate production were hypersusceptible to killing by the bacterial pathogens *P*. *aeruginosa*, *E*. *faecalis*, and *S*. *marcescens* in a manner dependent on oleate. Oleate is among the most abundant fatty acids in cells. Thus, these data may explain how a metazoan animal limits the induction of protective immune defenses to times when the host has accumulated sufficient energy reserves to survive challenge from bacterial pathogens.

Nandakumar et al. previously identified two fatty acids, GLA and SDA, which are synthesized by the enzyme *fat-3* and are required for the basal expression of immune effectors [17]. Our data indicate that oleate and these PUFAs affect immune effector expression and pathogen resistance by different mechanisms. *C*. *elegans* with a loss-of-function mutation in *fat-2*, the enzyme that catalyzes the first step in PUFA synthesis, induced innate immune effector genes normally and were not more susceptible to *P*. *aeruginosa* pathogenesis. It is also important to note that knockdown of *fat-3* did not affect the R24-mediated induction of 16 *fat-6*-dependent innate immune effectors. In addition, exogenous supplementation of PUFAs to *fat-6(RNAi)* animals did not restore immune effector expression, whereas the addition of oleate fully complemented the induction defect of these animals. Also of note, the effect of *fat-3(wa22)* on susceptibility to bacterial infection was independent of oleate.

Han et al. recently found that oleate is sufficient to extend the lifespan of nematodes that were grown under standard laboratory conditions [1]. Interestingly, we found that treatment with oleate is not sufficient to provide protection during bacterial infection, but is required for proper immune gene transcription. Specifically, oleate is important for the pathogen-mediated induction of immune effectors that are downstream of the p38 MAPK PMK-1 pathway and for genes that are regulated by the bZIP transcription factor ZIP-2, which functions independently of the canonical PMK-1 pathway to mediate an early transcriptional response to *P*. *aeruginosa* infection [31]. Consistent with these data, RNAi mediated knockdown of *tir-1*, a component of the p38 MAPK PMK-1 signaling cassette, as well as *zip-2*, enhanced the susceptibility of *fat-6(tm331);fat-7(wa36)* animals to *P*. *aeruginosa* infection. Thus, oleate has diverse, health-promoting effects on lifespan and pathogen resistance in *C*. *elegans*.

In plants, oleate is also required for the proper expression of immune defense genes and resistance to pathogen infection, suggesting that the role for oleate in immune activation may be strongly conserved [34,35]. However, the mechanism by which oleate regulates immune defenses is not known in either *C*. *elegans* or plants. Our supplementation studies indicate that oleate treatment itself does not activate immune gene transcription. Thus, oleate is unlikely to be a signal of immune activation in *C*. *elegans*, but rather functions as a licensing factor for the elaboration of anti-pathogen responses. Disruption of oleate biosynthesis alters membrane fluidity, which has pleiotropic consequences on membrane-bound organelles, including activating stress pathways associated with endoplasmic reticulum dysfunction [3]. Indeed, alterations of membrane fluidity have been linked to activation of G protein-coupled receptors [36,37]. Thus, changing the oleate content in *C*. *elegans* may modulate the ability of the host to mount protective defense responses, either directly or by disrupting lipid-protein interactions that are essential for immune pathway activation. Our findings present a previously unappreciated link between a highly abundant fatty acid and immune activation, which may represent an ancient connection between body energy stores and susceptibility to bacterial infection.

## Materials and methods

### *C*. *elegans* and bacterial strains

*C*. *elegans* strains were maintained on *E*. *coli* OP50 or HT115 bacteria on nematode growth media plates, as described [38]. The *C*. *elegans* strains used in this study were N2 Bristol [38], AU306 *agIs43* [*irg-4*::*GFP*::*unc-54-3’UTR; myo-2*::*mCherry*] [21], AY101 *acIs101* [p*DB09*.*1(irg-5*::*GFP)*; p*RF4(rol-6(su1006))*] [39], BX156 *fat-6(tm331);fat-7(wa36)* [9], BX106 *fat-6(tm331)* [5], BX153 *fat-7(wa36)* [5], BX30 *fat-3(wa22)* [6], and BX26 *fat-2(wa17)* [6]. *P*. *aeruginosa* strain PA14 [3], *E*. *faecalis* strain MMH594 [40], and *S*. *marcescens* strain Db11 [41] were used in this study.

### Fatty acid supplementation

Fatty acids were obtained from Nu-Chek-Prep, Inc. and were prepared as previously described [42]. Assays were performed by growing synchronized L1 worms on the indicated fatty acid or control media containing tergitol (0.1%). Unless otherwise indicated, oleate was used at a concentration of 400 or 500 μM, except for Fig 1E, which used 1 mM. Palmitoleic acid and linoleic acid were used at a concentration of 500 μM.

### *C*. *elegans* bacterial infection and other assays

“Slow killing” *P*. *aeruginosa* pathogenesis assays were performed as previously described [43]. The *E*. *faecalis* [40,44] and *S*. *marcescens* [45] pathogenesis assays were performed as previously described. For the assays with fatty acid supplementation, L4 stage-matched *C*. *elegans*, raised from the L1 to the L4 stage on *E*. *coli* HT115 on media containing fatty acids or control, were transferred to standard assay plates for the indicated experiment, which were not supplemented with fatty acids. Sample sizes, mean lifespan, and p values for all trials are shown in S2 Table. The protocol for treatment of animals with 70 μM R24 has also been described [22]. The RNAi screen of 1,420 RNAi clones was previously described [21]. RNAi clones that were used in this study are from the Ahringer [46] or Vidal [47] libraries and were confirmed by sequencing.

### NanoString nCounter gene expression analyses and quantitative RT-PCR (qRT-PCR)

The codeset used for the NanoString nCounter Gene Expression Analysis was synthesized by NanoString and contained probes for 118 *C*. *elegans* genes, which has been described previously [21,23]. Counts from each gene were normalized to three control genes: *snb-1*, *ama-1*, and *act-1*. The qRT-PCR studies were performed as described previously [21–23], using previously published primer sequences [8,15,21–23]. All values were normalized against the control gene *snb-1*. Fold change was calculated using the Pfaffl method [48].

### Gas chromatography and mass spectrometry (GC-MS)

Synchronized populations of approximately 6,000 worms at the L4 stage were harvested 24 hours after exposure to 70 μM R24 or 1% DMSO control, washed with M9 buffer to remove excess bacteria, and frozen in ethanol on dry ice. Worm pellets were thawed, sonicated, and then dissolved in 1 mL of a 3:1 methanol: methylene chloride mixture with 50 μl of internal standard dissolved in hexane (17:0, Nu-Chek-Prep Inc.). While vortexing, 200 μl acetyl chloride was slowly added. Samples were subjected to methanolysis at 80°C for 1 hour. After cooling to room temperature, the sample was neutralized with 4 mL of 7% K_2_CO_3_, and fatty acid methyl esters were extracted through the addition of 2 mL of hexane. Following hexane addition, samples were vortexed and then centrifuged at 2,500 rpm for 10 minutes. The top hexane layer containing fatty acid methyl esters was transferred to a new borosilicate glass test tube and washed with 2 mL acetonitrile, vortexed, and centrifuged at 2,500 rpm for 5 min. The top hexane layer was transferred and dried under nitrogen. Fatty acid methyl esters were resuspended in 200 μl hexane, vortexed, and transferred to Agilent vials with glass insert. Fatty acid methyl esters were analyzed by GC-MS using an Agilent 6890/5972 GC-MS system outfitted with a Supelcowax 10 column as previously described [49,50]. The relative abundance of each fatty acid was determined by dividing each fatty acid by the total fatty acid pool.

### Microscopy

Nematodes were paralyzed with 10 mM levamisole (Sigma), mounted on agar pads and photographed using a Zeiss AXIO Imager Z2 microscope with a Zeiss Axiocam 506mono camera and Zen 2.3 (Zeiss) software.

### Statistical analyses

*C*. *elegans* survival was assessed using the Kaplan-Meier method and differences were determined with the log-rank test using OASIS 2 [51]. Other statistical tests, which are indicated in the figure legends, were performed using Prism 7 (GraphPad Software).

## Supporting information

S1 Fig An RNAi screen identifies a role for MUFAs in the activation of *C*. *elegans* innate immune effectors. **A.** *C*. *elegans* carrying the *irg-4*::*GFP* immune reporter grown on control or *fat-7(RNAi)* bacteria and exposed to R24 or solvent control at the L4 stage. **B.** Wild-type *C*. *elegans* or *mdt-15(tm2182)* loss-of-function *C*. *elegans* grown on control media or media supplemented with oleate and exposed to R24 at the L4 stage. **C.** GC-MS of L4-stage animals grown on control or *fat-6(RNAi)* exposed to solvent control for 24 hours. Data are the average of three independent replicates with error bars showing standard deviation. Statistical analyses performed using two-way ANOVA with Bonferroni correction. * p<0.05.
(TIF)

S2 Fig *C*. *elegans fat-6(tm331)* and *fat-7(wa36)* mutants do not affect the R24-mediated induction of innate immune effector genes. qRT-PCR was used to assess the expression of the immune effector genes *irg-4*, *irg-5*, and *irg-6* in *fat-6(tm331)* (A) and *fat-7(wa36)* (B) animals exposed to the solvent control or R24 compared to wild-type. Data are the average of four independent replicates, each normalized to a control gene and presented as fold induction in R24-exposed vs. solvent control-exposed animals. Error bars show standard deviation.
(TIF)

S3 Fig *C*. *elegans fat-6(tm331)* and *fat-7(wa36)* mutants are not more susceptible to killing by *P*. *aeruginosa* than wild-type animals. *P*. *aeruginosa* pathogenesis assay of wild-type and the indicated mutant worms are presented. There is no significant difference between these conditions. Data are representative of two trials. Sample sizes, mean lifespan and p values for both trials are shown in S2B Table. Significance was determined using Kaplan-Meier survival curves and log-rank tests.
(TIF)

S4 Fig The effect of *fat-3(wa22)* on susceptibility to *P*. *aeruginosa* infection is independent of oleate. *P*. *aeruginosa* pathogenesis assays of wild-type and *fat-3(wa22)* animals grown on control media or oleate-supplemented media, as indicated. Animals were transferred at the L4 stage to plates containing *P*. *aeruginosa*. Assay plates were not supplemented with oleate. The *fat-3(wa22)* mutant is more susceptible to killing by *P*. *aeruginosa* (p<0.01). Oleate supplementation did not rescue the enhanced susceptibility to pathogens phenotype of the *fat-3(wa22)* mutant (p is not significant). Data are representative of two trials. Sample sizes, mean lifespan, and p values for all trials are shown in S2D Table. Significance was determined using Kaplan-Meier survival curves and log-rank tests.
(TIF)

S1 Table Relative expression of the 118 genes in the NanoString experiment reported in Fig 2. (XLSX)

S2 Table Sample sizes, mean lifespan, and p values for all trials of the *C*. *elegans* pathogenesis assays. **A.** All trials for the pathogenesis assays presented in Fig 4. **B.** All trials for the pathogenesis assays presented in S3 Fig. **C.** All trials for the pathogenesis assays presented in Fig 5. **D.** All trials for the pathogenesis assays presented in S4 Fig.
(XLSX)
