# Supplementary figures and images for: The fatty acid oleate is required for innate immune activation and pathogen defense in Caenorhabditis elegans
Source: PLoS Pathog. 2019 Jun 17;15(6):e1007893. doi: 10.1371/journal.ppat.1007893 (PMC6597122; doi:10.1371/journal.ppat.1007893)

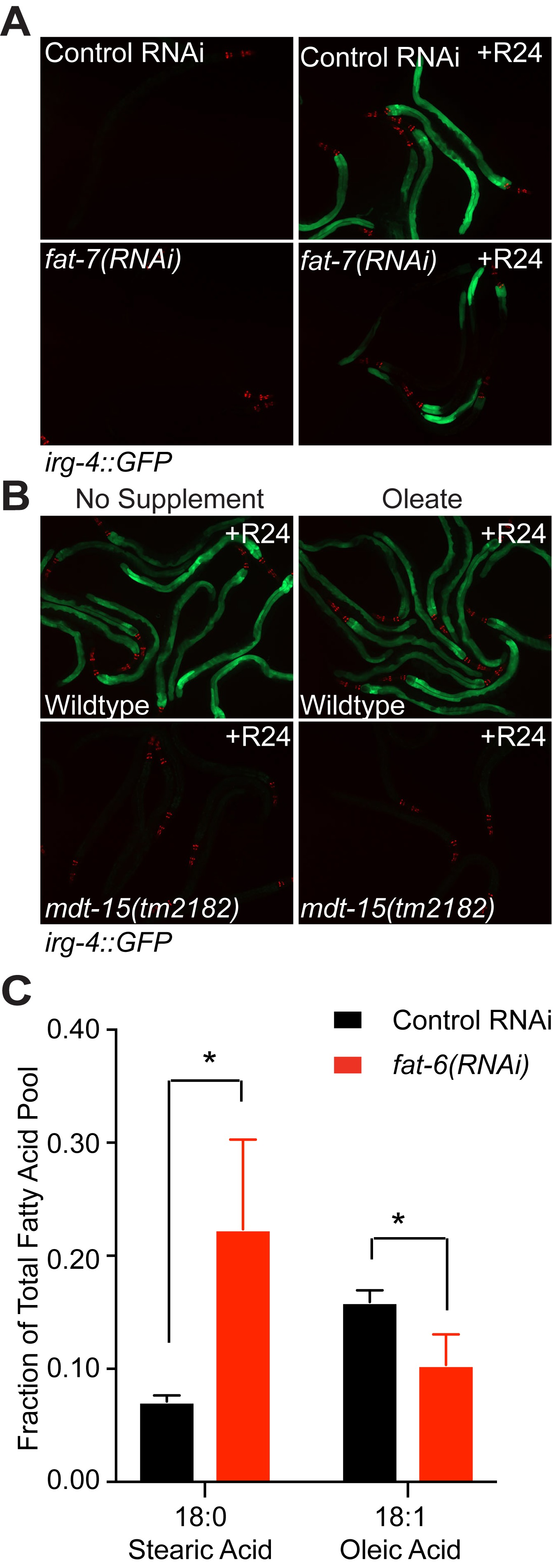

Supplement: S1 Fig — A. C. elegans carrying the irg-4::GFP immune reporter grown on control or fat-7(RNAi) bacteria and exposed to R24 or solvent control at the L4 stage. B. Wild-type C. elegans or mdt-15(tm2182) loss-of-function C. elegans grown on control media or media supplemented with oleate and exposed to R24 at the L4 stage. C. GC-MS of L4-stage animals grown on control or fat-6(RNAi) exposed to solvent control for 24 hours. Data are the average of three independent replicates with error bars showing standard deviation. Statistical analyses performed using two-way ANOVA with Bonferroni correction. * p<0.05. (TIF) [file ppat.1007893.s001.tif]

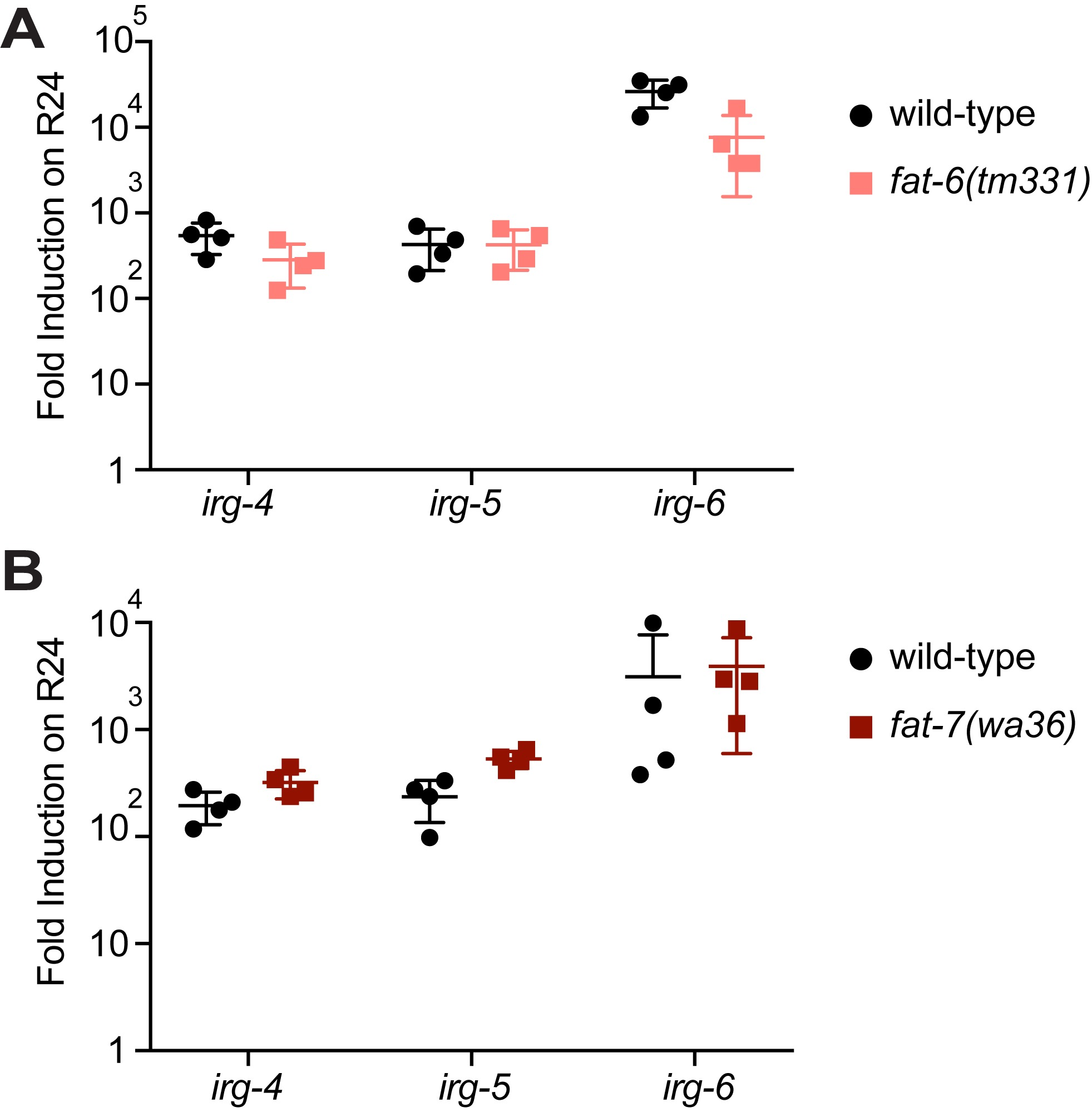

Supplement: S2 Fig — qRT-PCR was used to assess the expression of the immune effector genes irg-4, irg-5, and irg-6 in fat-6(tm331) (A) and fat-7(wa36) (B) animals exposed to the solvent control or R24 compared to wild-type. Data are the average of four independent replicates, each normalized to a control gene and presented as fold induction in R24-exposed vs. solvent control-exposed animals. Error bars show standard deviation. (TIF) [file ppat.1007893.s002.tif]

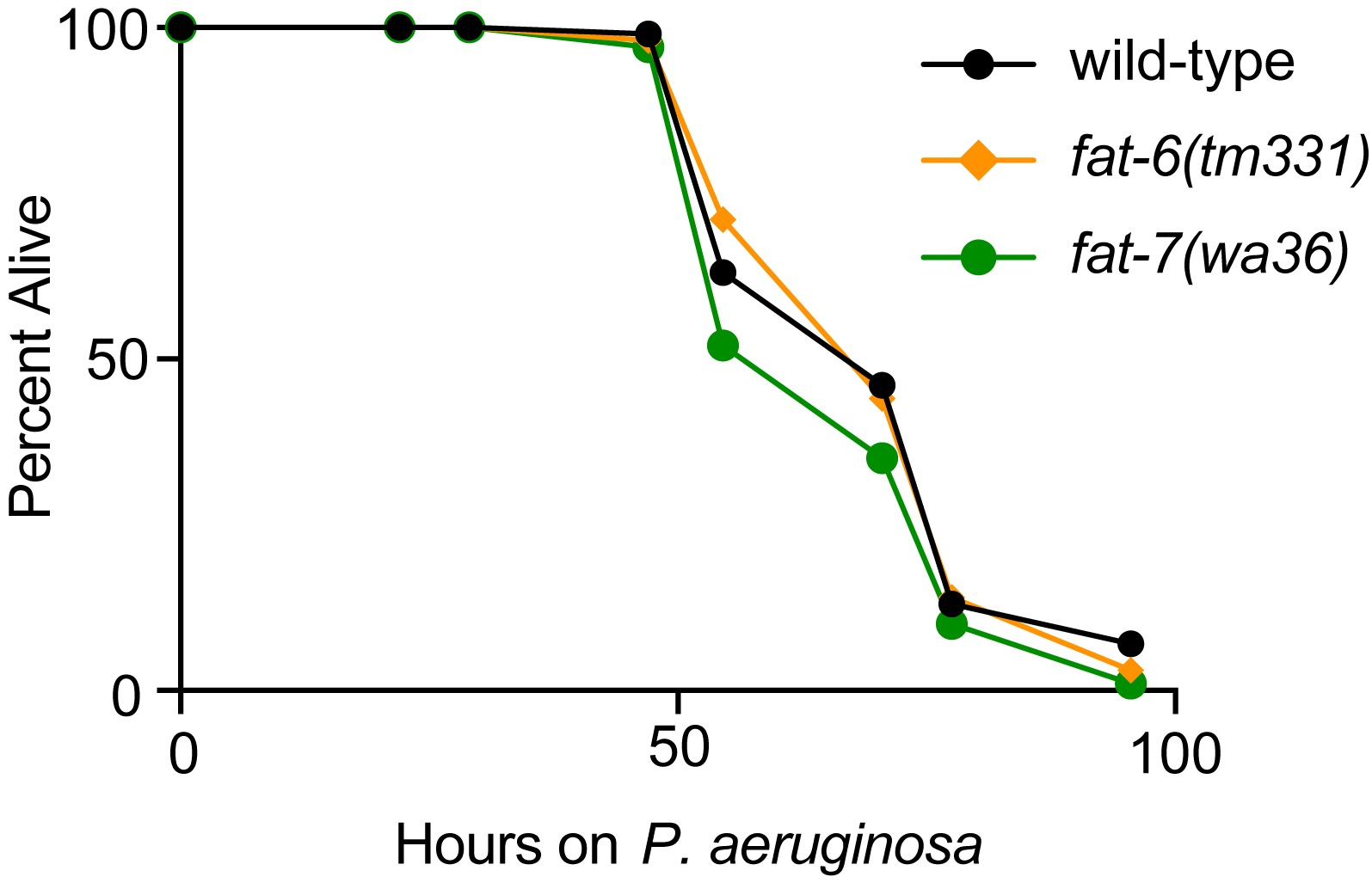

Supplement: S3 Fig — P. aeruginosa pathogenesis assay of wild-type and the indicated mutant worms are presented. There is no significant difference between these conditions. Data are representative of two trials. Sample sizes, mean lifespan and p values for both trials are shown in S2B Table. Significance was determined using Kaplan-Meier survival curves and log-rank tests. (TIF) [file ppat.1007893.s003.tif]

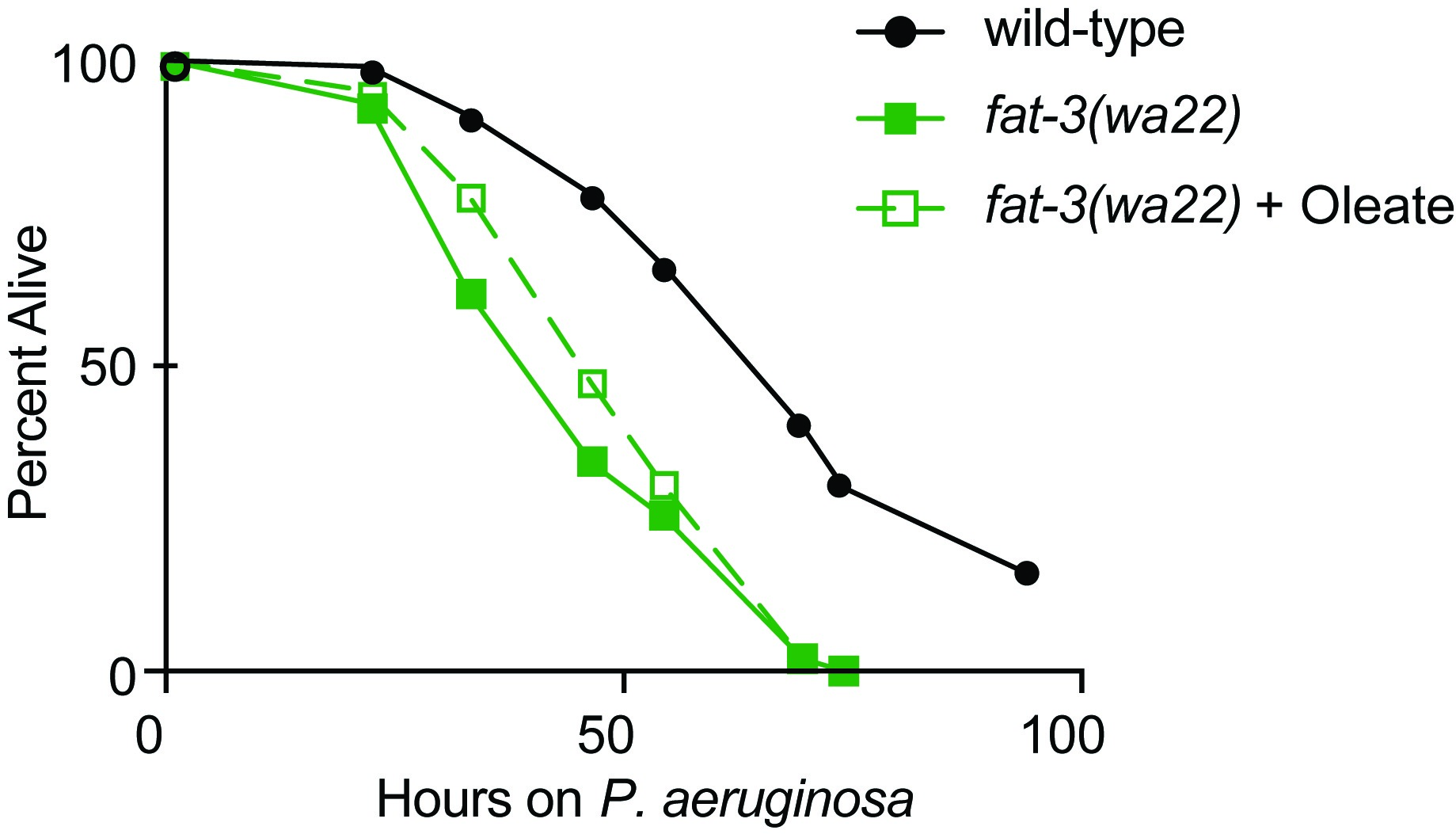

Supplement: S4 Fig — P. aeruginosa pathogenesis assays of wild-type and fat-3(wa22) animals grown on control media or oleate-supplemented media, as indicated. Animals were transferred at the L4 stage to plates containing P. aeruginosa. Assay plates were not supplemented with oleate. The fat-3(wa22) mutant is more susceptible to killing by P. aeruginosa (p<0.01). Oleate supplementation did not rescue the enhanced susceptibility to pathogens phenotype of the fat-3(wa22) mutant (p is not significant). Data are representative of two trials. Sample sizes, mean lifespan, and p values for all trials are shown in S2D Table. Significance was determined using Kaplan-Meier survival curves and log-rank tests. (TIF) [file ppat.1007893.s004.tif]
